# Regulation of Insulin and Leptin Signaling by Muscle Suppressor of Cytokine Signaling 3 (SOCS3)

**DOI:** 10.1371/journal.pone.0047493

**Published:** 2012-10-24

**Authors:** Zhenggang Yang, Matthew Hulver, Ryan P. McMillan, Lingzhi Cai, Erin E. Kershaw, Liqing Yu, Bingzhong Xue, Hang Shi

**Affiliations:** 1 Department of Internal Medicine, Wake Forest University School of Medicine, Winston-Salem, North Carolina, United States of America; 2 State Key Laboratory of Infectious Disease Diagnosis and Treatment, First Affiliated Hospital of Zhejiang University, Hang Zhou, China; 3 Department of Human Nutrition, Foods, and Exercise, Virginia Polytechnic Institute and State University, Blacksburg, Virginia, United States of America; 4 Division of Endocrinology, Diabetes, and Metabolism, University of Pittsburgh, Pittsburgh, Pennsylvania, United States of America; 5 Department of Animal and Avian Sciences, University of Maryland, College Park, Maryland, United States of America; 6 Department of Biology, Georgia State University, Atlanta, Georgia, United States of America; CRCHUM-Montreal Diabetes Research Center, Canada

## Abstract

Skeletal muscle resistance to the key metabolic hormones, leptin and insulin, is an early defect in obesity. Suppressor of cytokine signaling 3 (SOCS3) is a major negative regulator of both leptin and insulin signaling, thereby implicating SOCS3 in the pathogenesis of obesity and associated metabolic abnormalities. Here, we demonstrate that SOCS3 mRNA expression is increased in murine skeletal muscle in the setting of diet-induced and genetic obesity, inflammation, and hyperlipidemia. To further evaluate the contribution of muscle SOCS3 to leptin and insulin resistance in obesity, we generated transgenic mice with muscle-specific overexpression of SOCS3 (MCK/SOCS3 mice). Despite similar body weight, MCK/SOCS3 mice develop impaired systemic and muscle-specific glucose homeostasis and insulin action based on glucose and insulin tolerance tests, hyperinsulinemic-euglycemic clamps, and insulin signaling studies. With regards to leptin action, MCK/SOCS3 mice exhibit suppressed basal and leptin-stimulated activity and phosphorylation of alpha2 AMP-activated protein kinase (α2AMPK) and its downstream target, acetyl-CoA carboxylase (ACC). Muscle SOCS3 overexpression also suppresses leptin-regulated genes involved in fatty acid oxidation and mitochondrial function. These studies demonstrate that SOC3 within skeletal muscle is a critical regulator of leptin and insulin action and that increased SOCS may mediate insulin and leptin resistance in obesity.

## Introduction

Obesity, a disorder of impaired energy metabolism, is highly associated with insulin resistance and overt type 2 diabetes mellitus [1]. There is considerable evidence that energy balance and glucose homeostasis are physiologically regulated by the key metabolic hormones leptin and insulin, whose signaling network and ultimate action are interconnected in multiple tissues [1]. In addition, the development of resistance to the action of both leptin and insulin, which can occur with age, obesity and inflammation, appears to have a prime role in the pathogenesis of obesity, type 2 diabetes, and other metabolic disorders.

Skeletal muscle is a key metabolic tissue for insulin-stimulated glucose disposal and for energy metabolism. It represents about 40% of total body mass in nonobese subjects, and accounts for up to 30% of resting metabolic rate [2]. Skeletal muscle is a critical contributor to insulin resistance and obesity. Multiple studies have demonstrated that skeletal muscle-specific defects in insulin signaling contribute to systemic metabolic phenotypes [3], [4], [5]. While substantially less is known about the effects of leptin action in skeletal muscle, leptin has been shown to regulate lipid metabolism through direct activation of AMP-activated protein kinase (AMPK) [6], [7] in this tissue. The ability of leptin to stimulate muscle AMPK is significantly impaired in high fat diet-challenged mice, suggesting muscle leptin resistance in obesity [8]. Thus, insulin and/or leptin resistance in skeletal muscle may play a significant role in the pathogenesis of obesity and type 2 diabetes. Nevertheless, the cellular mechanisms linking insulin and leptin resistance in skeletal muscle remain poorly understood.

Suppressor of cytokine signaling 3 (SOCS3) is one of the SOCS protein family members, each of which contains a central SH2 domain and a conserved C-terminal SOCS box [1]. The SOCS proteins are rapidly induced by cytokines, and act as negative feedback regulators of cytokine signaling through several mechanisms, including direct binding to tyrosine-phosphorylated JAK or cytokine receptors and proteasomal degradation of signaling proteins via a SOCS box-mediated ubiquitination complex [1]. Previous work has established SOCS3 as a negative regulator of leptin signaling in hypothalamus [9], [10]. Leptin induces SOCS3, which in turn inhibits leptin signaling [9], [10], suggesting that SOCS3 plays a role in regulating leptin sensitivity. Previous studies have also shown that SOCS3 is a negative regulator of insulin signaling in fat and liver [11], [12], [13], [14]. Taken together, accumulating evidence strongly suggests that a single molecule, suppressor of cytokine signaling 3 (SOCS3), may mediate both leptin and insulin resistance due to its ability to inhibit leptin and insulin signaling in both central and peripheral target tissues [9], [10], [11], [12], [13]. However, the role of SOCS3 in skeletal muscle, a key tissue contributing to obesity and insulin resistance, is not known. We hypothesize that skeletal muscle SOCS3 contributes to obesity and insulin resistance by antagonizing leptin and insulin signaling**.** To test this hypothesis, we characterized muscle SOCS3 expression in response to metabolic challenges known to be associated with insulin and/or leptin resistance such as high-fat diet feeding, genetic obesity, lipid infusion, and TNFα injection. We then determine the metabolic consequences of transgenic overexpression of SOCS3 exclusively in skeletal muscle of mice, including evaluation of leptin and insulin signaling pathways.

## Results

### SOCS3 Expression is Elevated in Skeletal Muscle of Mice with Obesity, Inflammation and Hyperlipidemia

**Figure 1:**
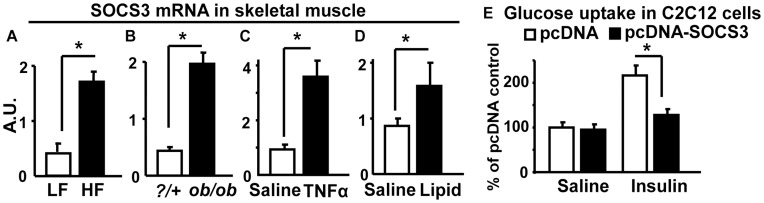
SOCS3 mRNA is elevated in skeletal muscle of diet-induced obese (A), *ob/ob* (B), TNFα-injected (C) and lipid-infused (D) mice. Total RNA was isolated from gastrocnemius and SOCS3 RNA levels were measured by quantitative real-time RT-PCR. (**E**). Over-expressing SOCS3 in C2C12 myotubes inhibits insulin-stimulated glucose uptake. C2C12 myotubes were treated with or without 100 nM insulin for 20 min. Data are expressed as mean ± SE, n = 6–8. *p<0.05. A.U.: arbitrary units.

Evaluation of SOC3 mRNA expression in murine skeletal gastrocnemius muscle under conditions known to be associated with insulin resistance revealed that SOC3 expression was increased by high-fat diet-induced obesity (DIO) (**Fig. 1A**), genetic obesity due to leptin deficiency (*ob/ob*) mice (**Fig. 1B**), and inflammation due to injection with the pro-inflammatory cytokine TNFα (**Fig. 1C**) and hyperlipidemia due to lipid infusion (**Fig. 1D**) [15], [16]. Similar increase, albeit to a lesser extent, was also observed in soleus of DIO and *ob/ob* mice (**Figure S1).** In addition, over-expressing SOCS3 in C2C12 myotubes suppressed insulin-stimulated glucose uptake (**Fig. 1E**). These data suggest that skeletal muscle SOCS3 is increased in conditions associated with obesity and insulin resistance, and may be an important molecule mediating obesity-, lipid-, and cytokine-induced insulin resistance.

### Creation of Transgenic Mice Over-expressing SOCS3 in Skeletal Muscle

**Figure 2:**
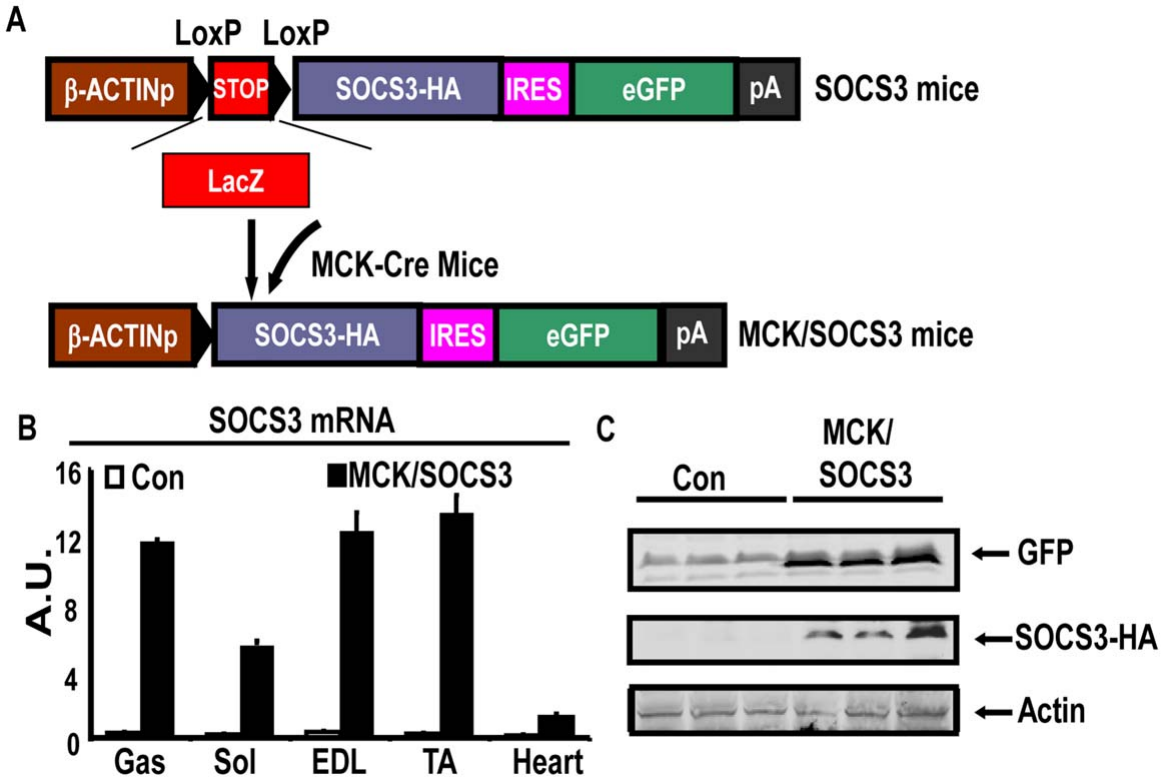
Creation of transgenic mice overexpressing SOCS3 in skeletal muscle. (**A**) The SOCS3 transgene construct. Transgenic mice (SOCS3) were crossed with MCK-Cre mice to generate mice over-expressing SOCS3 specifically in skeletal muscle (SOCS3/MCK). (**B**) SOCS3 mRNA is increased in SOCS3/MCK mice compared to SOCS3 mice. SOCS3 mRNA in various types of skeletal muscle was measured by real-time RT PCR. Gas: gastrocnemius; Sol: soleus; EDL: extensor digitorum longus; TA: tibialis anterior. n = 4. (C) The tag protein GFP and the transgene-encoded protein SOCS3-HA are expressed in gastrocnemius skeletal muscle. The proteins were detected by immunoblotting using specific antibodies.

To determine whether up-regulation of muscle SOCS3 expression contributes to the development of obesity and insulin resistance, we generated a transgenic murine model for over-expressing SOCS3 in tissue specific manner. The SOCS3 construct is shown in **Fig. 2A** and is described in Material and Methods and references [17], [18], [19]. By PCR genotyping of LacZ transgene and Xgal staining, we identified 32 transgenic founders, two of which showed increased SOCS3 expression in skeletal muscle after crossing with the MCK-Cre mouse expressing Cre recombinase specifically in muscle under the control of the MCK promoter [20]. The line 8 mice, when crossed with MCK Cre mice, had a 20–30 fold increase of SOCS3 mRNA in all types of muscle (eg, gastrocnemius (Gas), soleus (Sol), extensor digitorum longus (EDL) and tibialis anterior (TA)), designated as the MCK/SOCS3 mice, compared with the control SOCS3 mice without the presence of the MCK Cre (designated as control mice) (**Fig. 2B**), whereas no differences in SOCS3 expression have been detected in other tissues including hypothalamus, fat, liver, pancreas, kidney and spleen etc. (data not shown). In addition, we also detected the expression of the transgene protein by immunoblotting eGFP and HA tag with specific antibodies (**Fig. 2C**). Thus, MCK/SOCS3 mice have increased SOC3 mRNA and protein expression exclusively in skeletal muscle.

### Over-expression of SOCS3 Blocks Insulin Signaling in Skeletal Muscle

To determine the effect of muscle-specific SOCS3 overexpression on overall energy homeostasis, we determine body weight in response to low fat (LF) or high fat (HF) feeding. MCK/SOCS3 mice and control mice displayed similar body weights (**Figure. S2A**). There was no difference in food intake between MCK/SOCS3 and control mice (**Figure S2B**).

**Figure 3:**
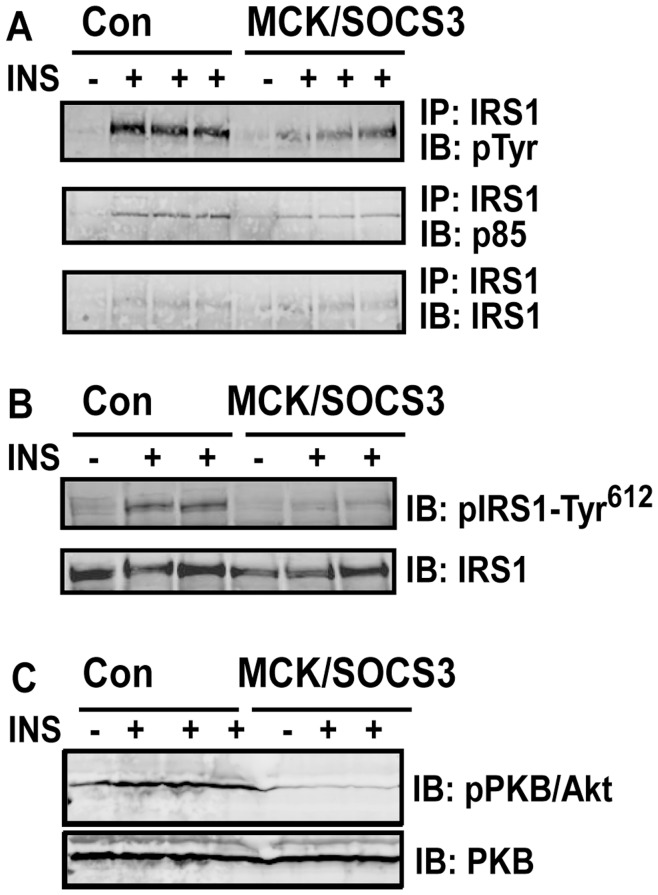
Skeletal muscle SOCS3 over-expression blocks insulin signaling in skeletal muscle. 5-month old mice on chow diet were intraperitoneally injected with 10 units/kg body weight of human insulin or saline. 10 min after injection, gastrocnemius muscle strips were dissected and saved for immunoprecipitation and immunoblotting analysis of insulin signaling molecules as described in Materials and Methods.

Previous data have established that SOCS3 is a negative regulator of insulin signaling in fat and liver [11], [12], [13], [14]. However, the role of SOCS3 in regulating muscle insulin signaling is not known. We therefore examined muscle insulin signaling in MCK/SOCS3 and control mice fed a chow diet. Since IRS-1 is the major signaling protein that SOCS3 targets to inhibit insulin signaling [12], [13], we first measured insulin-stimulated IRS-1 phosphorylation. As shown in **Fig. 3A and 3B**, insulin injection markedly stimulated the overall tyrosine and specific tyrosine (Tyr612) phosphorylation of insulin receptor substrate 1 (IRS-1) in gastrocnemius muscle of control mice, while this signaling event was attenuated in MCK/SOCS3 mice. p85 is a regulatory subunit of PI 3-kinase, and p85 docking to IRS proteins is essential for regulation of PI-3 kinase activity. We therefore evaluated the docking of p85 to IRS-1 in the skeletal muscle of MCK/SOCS3 and control mice. IRS-1 was immunoprecipitated, followed by immunoblotting with the p85 antibody. **Fig. 3A** shows that insulin-stimulated association of p85 with IRS-1 was reduced in MCK/SOCS3 mice, suggesting an attenuation of PI-3 kinase activity by muscle SOCS3 over-expression. Similar results were observed on the effect of SOCS3 over-expression on Akt phosphorylation (**Fig. 3C**). These data suggest that SOCS3 over-expression impairs local insulin signaling in skeletal muscle.

### Muscle SOCS3 Over-expression Causes Systemic Insulin Resistance

**Figure 4:**
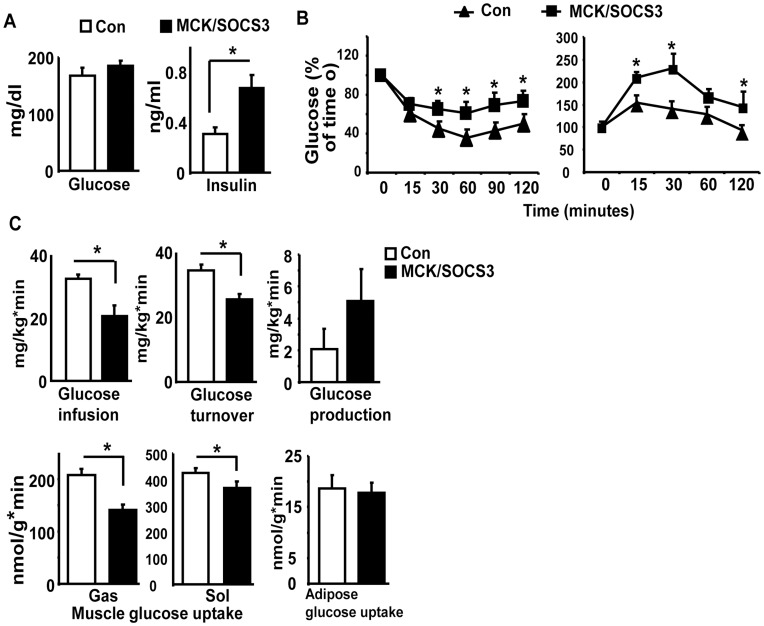
Skeletal muscle SOCS3 over-expression impairs systemic insulin sensitivity. (**A**) Blood glucose levels (left panel) and blood insulin levels (right panel) in MCK/SOCS3 and control mice fed a low fat chow diet. (**B**): ITTs (left) and GTTs (right) in MCK/SOCS3 and control mice. ITTs and GTTs were performed on mice fed a chow diet as described in Materials and Methods. (**C**) Glucose infusion rate, insulin-stimulated glucose turnover, hepatic glucose production under clamp, and insulin-stimulated glucose uptake in gastrocnemius/soleus muscle and epididymal fat. Hyperinsulinemic-euglycemic clamps were conducted in MCK/SOCS3 and control mice fed a chow diet as described in Materials and Methods. Data are expressed as mean ± SE; n = 5, *p<0.05 vs. control SOCS3 mice.

To determine whether impaired muscle insulin signaling caused by SOCS3 over-expression can be translated into systemic insulin resistance, we characterized the whole body insulin sensitivity of MCKSOCS3 mice fed a chow diet. Although there was no difference in fed glucose levels between control and MCK/SOCS3 mice, MCK/SOCS3 mice had higher fed insulin levels, suggesting that MCK/SOCS3 mice exhibited insulin resistance (**Fig. 4A**). To further confirm this finding, we performed ITTs and GTTs on MCK/SOCS3 and control mice. MCK/SOCS3 mice showed a lesser hypoglycemic response to intraperitoneal injection of insulin, compared with control mice (**Fig. 4B**). Likewise, MCK/SOCS3 mice were also more glucose intolerant in GTTs. In addition, we performed hyperinsulinemic-euglycemic clamp studies to examine the insulin sensitivity of glucose metabolism in MCK/SOCS3 and control mice. During a continuous insulin infusion at 2.5 mU/kg/min, the plasma insulin levels were increased to over 500 pM, and plasma glucose levels were maintained at ∼7 mM in both genotypes of mice (**Table S1**). The glucose infusion rate required to maintain euglycemia was reduced by 38% in MCK/SOCS3 mice compared with control mice (**Fig. 4C**). Similarly, insulin-stimulated glucose turnover was decreased by 27% in MCK/SOCS3 mice (**Fig. 4C**). Although there was a trend of increased hepatic glucose production in MCK/SOCS3 mice during the clamp stage, it did not reach statistical significance (**Fig. 4C**). Consistent with the measures of whole-body glucose turnover, MCK/SOCS3 mice displayed a 37% decrease in insulin-stimulated glucose uptake in gastrocnemius muscle, and to a lesser extent, a 13% decrease in soleus muscle, while there was no change in glucose uptake in epididymal fat between two genotypes (**Fig. 4C**). These data suggest that over-expression of SOCS3 in skeletal muscle causes systemic and tissue-specific insulin resistance.

### Skeletal Muscle Overexpression of SOCS3 Suppresses Basal AMPK Signaling

**Figure 5:**
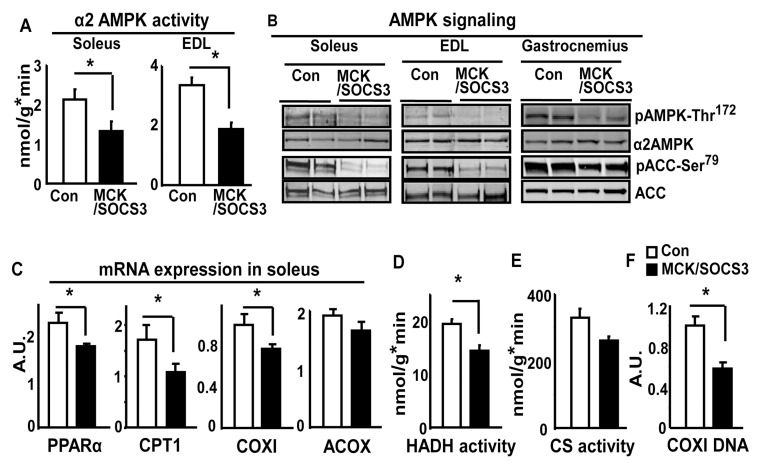
Mice with skeletal muscle over-expression of SOCS3 have suppressed basal AMPK signaling. (**A**) α2AMPK activity, (**B**) AMPK signaling, (**C**) expression of genes involved in fatty acid oxidation, (**D**) β-hydroxyacyl-CoA dehydrogenase activity (HADH), (**E**) citrate synthase activity (CS), and (**F**) COXI DNA content in skeletal muscle of SOCS3/MCK and control mice on a chow diet. Soleus, EDL or gastrocnemius muscles from 5-month-old chow-fed male mice were evaluated for AMPK activity using an immune complex assay. Phosphorylation and total protein levels of AMPK and ACC were measured by immunoblotting. Gene expression was measured by Real-time RT-PCR and corrected with cyclophilin levels. HADA and CS activities were determined spectrophotometrically using skeletal muscle (gastrocnemius) homogenates. Mitochondrial DNA content was measured in muscle (gastrocnemius) DNA samples by Real-time PCR using primer/probe sets corresponding to mitochondrial DNA-encoded cytochrome c oxidase subunit I (COXI) gene and normalized with nuclear DNA-encoded gene UCP2. Data are expressed as Mean ± SE, n = 6–8. *p<0.05 vs. control SOCS3 mice in (**A**), (**C**), (**D**), and (**F**); p = 0.05 vs. control SOCS3 mice in (**E**).

To determine the role of SOCS3 in regulation of leptin signaling in skeletal muscle, we examined the muscle AMPK pathway, a key cellular signal activated by leptin [21]. **Fig. 5A** shows that α2AMPK activity in soleus and EDL muscle of MCK/SOCS3 was decreased by 35%, compared with that of control mice. Consistent with these findings, AMPK phosphorylation was down-regulated in soleus, EDL and gastrocnemius muscle of SOCS3/SOCS3 mice (**Fig. 5B** **).** In addition, the phosphorylation of ACC, downstream target of AMPK and important regulator of fatty acid oxidation, was also decreased (**Fig. 5B**). These results were confirmed by quantitation of these blots (**Figure S3**). These data strongly suggest an inhibitory effect of SOCS3 on basal AMPK-ACC signaling pathway. Activation of AMPK results in up-regulation of genes involved in fatty acid oxidation and mitochondrial function. We determined whether SOCS3 over-expression antagonizes this action in skeletal muscle of transgenic mice. We found that SOCS3 over-expression decreased mRNA expression of such genes, including carnitine palmitoyl transferase 1 (CPT1), PPARα, cytochrome C oxidase I (COXI), and acyl-CoA oxidase (ACOX), in soleus muscle (**Fig. 5C**). In parallel, the activity of β-hydroxyacyl-CoA dehydrogenase (HADA), an enzyme involved in fatty acid oxidation, was decrease by 25% in MCK/SOCS3 mice (**Fig. 5D**). Consistent with this, the activity of citrate synthase (CS), a mitochondrial enzyme, was decreased by 20% in these transgenic mice, suggesting a suppression of mitochondrial enzymatic activity (**Fig. 5E**). We also examined the muscle mitochondrial content by measuring mitochondrial DNA-encoded gene COXI, an index that has been used to determine mitochondrial content in our previous publications [22], [23]. We found that the COXI DNA amount was decreased by 40% in soleus muscle of MCK/SOCS3 mice (**Fig. 5F**), suggesting a decrease in muscle mitochondrial content. Despite these observations, we were unable to detect a difference in muscle morphology or intramyocellular neutral lipid content under these conditions using histological methods (**Figure S4**). Thus, this SOCS3-mediated reduction in AMPK action may not be sufficient to impair overall intramyocellular lipid homeostasis perhaps due to compensatory mechanisms.

### Skeletal Muscle Overexpression of SOCS3 Blocks Leptin Activation of AMPK Signaling

**Figure 6:**
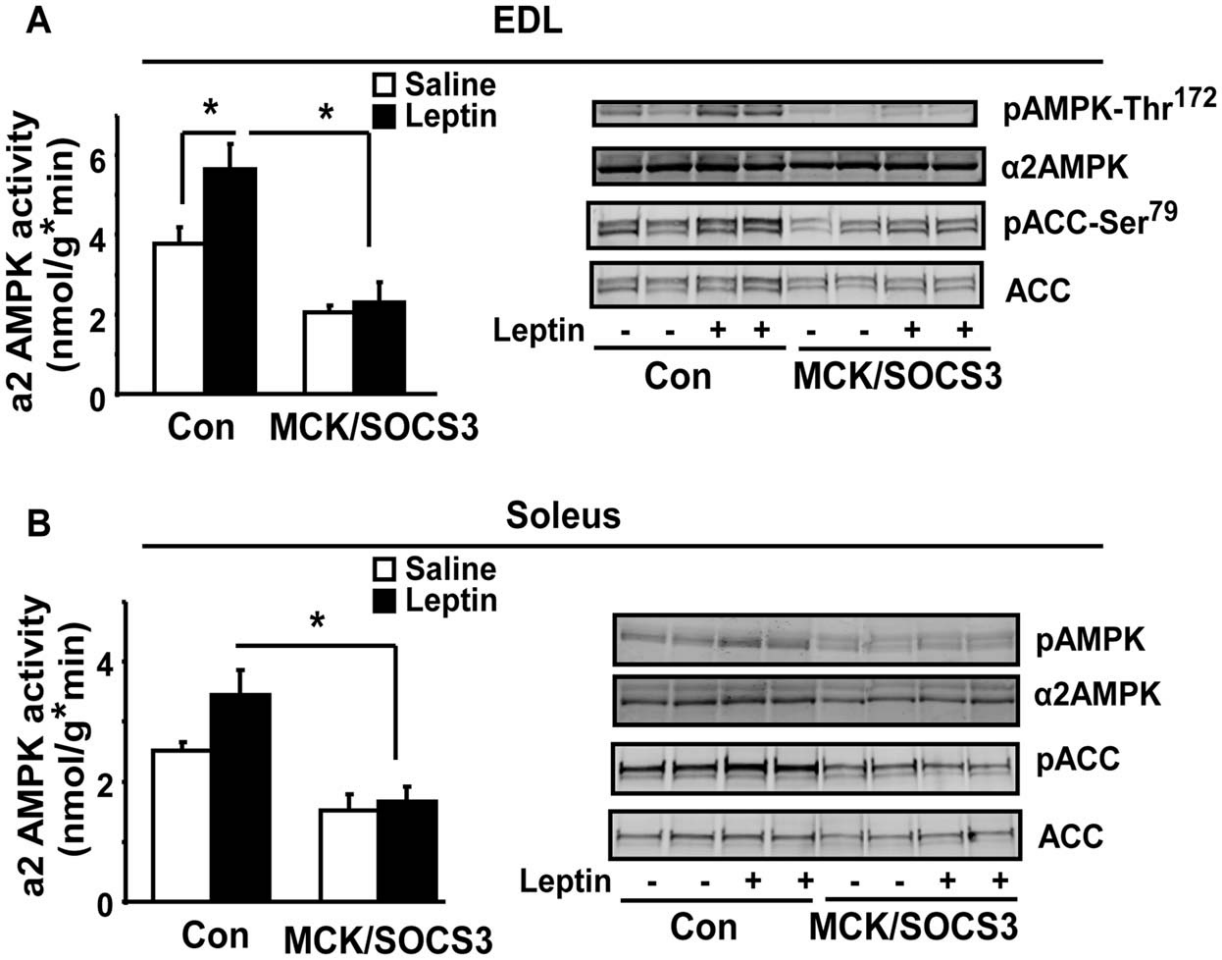
Skeletal muscle SOCS3 over-expression blocks leptin-activated AMPK signaling in EDL (A) and soleus (B) muscle. 5-month old mice on chow diet were intraperitoneally injected with leptin (3 mg/kg body weight) and euthanized 30 minutes later. Soleus and EDL muscle strips were quickly removed and frozen in liquid nitrogen. α2AMPK activity and AMPK and ACC phosphorylation were conducted as described in Figure 3. Data are expressed as Mean ± SE, n = 6–8. *p<0.05.

Since leptin can directly activate AMPK signaling in skeletal muscle [6], [7], we examined leptin-activated AMPK signaling in skeletal muscle of MCK/SOCS3 mice with muscle SOCS3 over-expression. In EDL muscle, Leptin injection for 30 min stimulated α2AMPK activity by 50% in control SOCS3 mice, while this stimulation was completely blocked in MCK/SOCS3 mice (**Fig. 6A** left). Similar results were observed in leptin-induced phosphorylation of AMPK at Thr172. Leptin injection resulted in phosphorylation of AMPK Thr172 in EDL of control mice, but not MCK/SOCS3 mice (**Fig. 6A** right). Consistent with these findings, leptin-induced phosphorylation of ACC was also attenuated in EDL of SOCS3/MCK mice, compared with control mice (**Fig. 6A** right). We further examined leptin-activated AMPK signaling in soleus muscle. SOCS3 over-expression in soleus exerted a similar inhibitory effect on both basal and leptin-stimulated AMPK signaling, although leptin’s ability to activate AMPK pathway was less potent in soleus compared with EDL (**Fig. 6B**). These data suggest that SOCS3 overexpression in muscle impairs leptin-mediated activation of α2AMPK and its downstream targets.

## Discussion

SOCS3 is best known for its negative regulation of leptin signaling and action in hypothalamus [9], [10], [24]. We and others have also shown that SOCS3 is a negative regulator of insulin signaling in fat and liver [11], [12], [13], [14]. Therefore, SOCS3 is a single molecule that mediates both leptin and insulin resistance due to its ability to down-regulate leptin and insulin signaling. These data are the first to demonstrate that SOCS3 antagonizes both leptin and insulin signaling in skeletal muscle, which is a principal site of glucose and fatty acid use and is one of the primary tissues responsible for insulin resistance in obesity [5]. Muscle SOCS3 is therefore positioned to play an important role in the pathogenesis of obesity-induced insulin resistance and type 2 diabetes by antagonizing both leptin and insulin signaling in skeletal muscle.

Skeletal muscle is the primary site of insulin-dependent glucose disposal and resistance of skeletal muscle to insulin’s action is an early step in the development of type 2 diabetes [3], [4], [5]. Despite the pivotal role of skeletal muscle in the development of systemic insulin resistance, the molecular mechanisms underlying skeletal muscle insulin resistance in the setting of obesity still remain elusive. Our study suggests that SOCS3 within skeletal muscle is a critical factor in the development on of both skeletal muscle-specific and systemic insulin resistance in response to obesity. MCK/SOCS3 mice develop impaired systemic and muscle-specific glucose homeostasis and insulin action evident in glucose and insulin tolerance tests, hyperinsulinemic-euglycemic clamps, and insulin signaling studies. Although the MCK/SOCS3 mice showed normal glycemia on regular low fat diets, they did exhibit hyperinsulinemia. Given the fact that these mice are normoglycemic despite insulin resistance, we speculate that they have higher insulin levels due to compensatory secretion of insulin from pancreas, similar to liver insulin receptor knockout (LIRKO) mice [25]. We previously generated a transgenic model over-expressing SOCS3 in adipose tissue. Although over-expression of SOCS3 in adipose tissue causes local insulin resistance, it is not sufficient to cause systemic insulin resistance, maybe due to the limited contribution of adipose tissue in whole-body glucose disposal. In contrast, our current genetic model with muscle SOCS3 over-expression demonstrates a prominent role for muscle SOCS3 in the development of systemic insulin resistance in obesity.

It has been demonstrated that SOCS3 negatively regulates insulin signaling in adipose tissue and liver [13], [14], [26] via several mechanisms, including binding to the IR at phosphorylated tyrosine 960 [27], inhibition of IRS-1 and IRS-2 tyrosine phosphorylation [14], proteosomal degradation of SOCS-associated protein (e.g. IRS-1) [12], and inhibition of Jak [27]. In this genetic model, SOCS3 appears to mainly antagonize IRS-1 phosphorylation. It is noteworthy that, in addition to the direct inhibition of SOCS3 on IRS-1 phosphorylation, other mechanisms might be involved in the development and complexity of the insulin resistant phenotype in our genetic model. Since activation of AMPK increases basal and insulin-stimulated glucose uptake in skeletal muscle skeletal muscle [28], SOCS3 may affect insulin signaling indirectly by antagonizing muscle AMPK signaling.

Although leptin is best known for its regulation of food intake and energy homeostasis via actions through neurons in the hypothalamus [29], increasing evidences demonstrate that leptin directly regulates lipid metabolism in extra-neural tissues, notably, skeletal muscle [30], [31]. Adenoviral-mediated leptin gene transfer in rats results in rapid depletion of triacylglycerol in tissues including skeletal muscle [32], [33], which is exerted at least in part via direct action of leptin in peripheral tissues [34]. Skeletal muscle expresses the long-form leptin receptor [35], and leptin directly activates lipid metabolism in skeletal muscle isolated from mice, humans, rats and C2C12 myotubes [6], [7], [36], [37], [38], [39], [40]. In addition, recent data show that leptin’s direct effects on skeletal muscle is mediated by activation of the AMPK signaling pathway [6], [7], which may involve up-regulation of genes responsible for fatty acid oxidation, such as peroxisome proliferator-activated receptor α (PPARα) [7]. However, the ability of leptin to stimulate muscle AMPK is significantly impaired in high fat diet-challenged mice, suggesting an early sign of leptin resistance [8]. Moreover, leptin stimulation of muscle fatty acid oxidation is significantly suppressed in obese humans and rodents [38], [39], which occurs as early as 4 weeks after high fat diet feeding [38]. These observations suggest that skeletal muscle leptin resistance, similar to central leptin resistance, is an early defect of, and may thereby play an important role in obesity and insulin resistance. Therefore, skeletal muscle leptin signaling, which may be mediated at least in part through direct activation of AMPK signaling, may be an important regulator of muscle lipid metabolism and resistance to leptin’s action in skeletal muscle may contribute to the development of obesity and insulin resistance. One prominent question is what signal(s) mediates muscle leptin resistance. Strong evidence from our current study points to SOCS3.

The roles of SOCS3 in antagonizing leptin’s effects and determining leptin resistance in hypothalamus have been relatively well characterized. Recent data suggest that leptin rapidly induces SOCS3 expression in L6 rat skeletal muscle cells [41]. In addition, skeletal muscle leptin resistance appears to be associated with increased expression of SOCS3, and over-expressing SOCS3 in primary human myotubes significantly inhibits leptin’s direct effect on AMPK-ACC signaling [42]. These data suggest that skeletal muscle SOCS3 may also be an important determinant of leptin resistance. Our study using more compelling genetic approaches demonstrates that skeletal muscle SOCS3 is a negative regulator of basal and leptin-activated α2AMPK signaling and downstream targets. We also observed a trend of increase of circulating leptin levels in transgenic mice with muscle SOCS3 over-expression (data not shown), similar to the scenario of insulin resistance where there is a compensatory increase of circulating insulin levels due to impaired insulin signaling in insulin sensitive tissues. Therefore, skeletal muscle SOCS3 may serve as a mediator of muscle leptin resistance. One limitation of our present study is that we did not measure α1AMPK activity and phosphorylation at either basal or leptin stimulated levels in our genetic model. Although α2AMPK is the major isoform that appears to be regulated by leptin signaling in skeletal muscle [6], α1AMPK can also be regulated in skeletal muscle in various physiological conditions [43]. Therefore, we can not rule out that α1AMPK may also be affected by SOCS3 expression and contribute to the metabolic phenotypes observed in our genetic mouse. Moreover, additional studies are required to address the molecular mechanisms whereby SOCS3 antagonizes AMPK signaling.

In summary, we demonstrate that muscle-specific over-expression of SOCS3 impairs muscle insulin signaling and promotes systemic insulin resistance. Furthermore, muscle-specific over-expression of SOCS3 suppresses the basal AMPK pathway and blocks the effect of leptin on AMPK activation. Thus, muscle-specific overexpression of SOCS3 is sufficient to negatively regulate both insulin and leptin action in skeletal muscle. These findings, in combination with early impairment in these pathways and increased SOC3 expression in muscle in obesity-associated metabolic states, strongly support a role for SOCS3 in the pathogenesis of these disorders.

## Materials and Methods

### Animals

The murine SOCS3 cDNA with a 3′ sequence encoding two HA tags was isolated from pcDNA 3.0 SOCS3 expression vector [9], blunt-ended and ligated into the Xho I site of the pCCALL2-IRES-EGFP vector previously used for transgene constructs described in references [17], [18], [19] (obtained through Dr. Brad Lowell’s lab, Beth Israel Deaconess Medical Center, Boston). Key features of the construct include the following: 1) a chicken β-actin promoter allowing ubiquitous expression of cDNA construct placed behind it; 2) a loxP-floxed transcriptional blocker that contains the *lacZ* gene, 3) coding sequence for SOCS3 with HA-tag, followed by ires-eGFP (internal ribosomal entry site-enhanced green fluorescent protein). With this design, the β-actin promoter can only drive SOCS3 expression when the transcriptional block is removed in the presence of Cre, therefore achieving tissue-specific over-expression. The SOCS3 construct was linearized with Stu I and injected into pronuclei of fertilized eggs from FVB mice in the Transgenic Core Facility at Beth Israel Deaconess Medical Center, Boston. 32 positive transgenic founders were obtained based on both PCR genotyping of LacZ transgene (Forward primer: 5′-gacgtctcgttgctgcataa-3′; Reverse primer: 5′-cagcagcagaccattttcaa-3′) and Xgal staining. After crossing with the MCK-Cre mouse expressing Cre recombinase specifically in muscle under the control of the muscle creatine kinase (MCK) promoter (Jackson Laboratory, Bar Harbor, ME), two independent lines exhibited increased SOCS3 expression in skeletal muscle and the line (line 8) with higher expression was used for further characterization and designated as MCK/SOCS3 mice.

The animal studies were approved by the institutional animal care and use committee of the Baptist Medical Center at Wake Forest University. SOCS3 mice were crossed with MCK-Cre mice to generate mice over-expressing SOCS3 in skeletal muscle (designated as SOCS3/MCK mice). The offsprings were screened by both PCR genotyping and Xgal staining. In the high-fat (HF) diet study, 5-week old SOCS3 mice and SOCS3/MCK mice were placed on either a low fat (LF) (Research Diets D12450B, 10% calories from fat, Research Diets Inc., New Brunswick, NJ) or a high fat (HF) diet (Research Diets D12492, 60% calories from fat, Research Diets Inc.) for 24 weeks. Body weight was measured weekly. Blood glucose was measured with an OneTouch Ultra Glucose meter (Lifescan, Milpitas, CA). Serum insulin levels were measured using rat insulin enzyme-linked immunosorbent assay (ELISA) kits (Crystal Chem, Downers Grove, IL). Insulin tolerance tests (ITTs) were performed as previously described [44]. Mice were injected intraperitoneally (ip) with 1.2unit/kg of human insulin (Humulin R, Eli Lilly, Indiana, IN) after a 6-hr fasting, and glucose levels were measured at different time points (0, 15, 30, 60, 120 minutes). For glucose tolerance tests (GTTs), mice were fasted overnight, and blood glucose was measured immediately before and 15, 30, 60, 120 minutes after ip injection of glucose (0.75 g/kg of body weight). After 24 weeks of LF/HF diet feeding, all mice were euthanized and blood was collected. Various fat pads (epididymal, subcutaneous, mesenteric and peri-renal) and skeletal muscles (gastronemius, soleus, extensor digitorum longus, and tibialis anterior) were dissected and frozen in liquid nitrogen and stored at −80°C.

For the study of HF diet feeding on muscle SOCS3 expression, 6-week-old male C57BL/6J mice were purchased from the Jackson Laboratory (Bar Harbor, ME), and were fed LF chow or HF diets (D12331, fat content 58% by calorie, Research Diets Inc.) for 16 weeks. For lipid infusion studies, 3-month-old male C57BL/6J mice were implanted with indwelling catheters (Micro-Renathane tubing, MRE-025; Braintree Scientific Inc., Braintree, MA). After a 5-day recovery, mice were infused with lipids (5ml/kg/hr; Liposyn II; Abbott) and heparin (6 U/hr) or saline for 8 hrs with a microdialysis pump (CMA/102; CMA Microdialysis). For TNFα injection experiments, 2-month-old male mice were injected with TNFα (3.3 µg). 10-week-old ob/ob mice were purchase from the Jackson Laboratory. After the respective treatments (HF feeding, lipid infusion or TNFα injection), mice were euthanized, and skeletal muscles (gastrocnemius, soleus, and EDL) were dissected and frozen in liquid nitrogen.

### Cell Culture and Glucose Transport Assay

C2C12 myoblasts were purchased from ATCC and cultured in DMEM containing 10% of FBS. Cells were transfected with pcDNA 3.0 SOCS3 expression vector [9] using a SuperFect Transfection Reagent kit (Qiagen, Valencia, CA). Cells were grown to confluence and cultured in DMEM containing 2% of horse serum to induce differentiation into myotubes. For glucose transport assay, C2C12 myotubes were incubated in KRH buffer (129 mM NaCl, 4.8 mM KCl, 1.2 mM MgSO_4_, 1.8 mM CaCl_2_, and 20 mM HEPES, PH 7.4) with or without 100 nM insulin for 20 min. To start the glucose transport, 1 µCi of 2-Deoxy-^3^H-glucose with cold deoxy-glucose was added to each well and incubated for 10 min. To stop the transport, cells were rinsed with ice-cold KRH buffer for 3 times and lysed with 0.1% SDS. Cell lysates were added to scintillation vials with 5 ml scintillation liquid and counted.

### Immunoblotting and Immunoprecipitation

For immunoblotting, frozen muscle tissues were homogenized in a modified radioimmunoprecipitation assay (RIPA) lysis buffer containing 50 mM Tris-HCL, 1 mM EDTA, 1% NP-40, 0.25% sodium deoxycholate, 150 mM NaCl, 1 mM PMSF, 200 µM Na_3_VO_3_, 1% protease inhibitor cocktail (Sigma), and 1% phosphatase inhibitor cocktail (Sigma). Tissue homogenates were incubated on ice for 45 min to solubilize all proteins, and insoluble portions were removed by centrifugation at 14,000 g at 4°C for 15 min. Proteins from whole tissue lysates were separated by SDS-polyacrylmide gel electrophoresis (PAGE) and the gels were transferred to nitrocellulose membrane (Bio-Rad, Hercules, CA). The transferred membranes were blocked, washed, incubated with various primary antibodies overnight at 4°C, and followed by fluorescence-conjugated secondary antibodies Alexa Fluor 680 (Invitrogen, Carlsbad, CA) at room temperature for 2 hrs. The blots were developed with a Li-COR Odyssey Infrared Imager system (Li-COR Biosciences). For immunoprecipitation, 1 mg of muscle lysate was incubated overnight with appropriate antibodies and protein A agarose (Santa Cruz) at 4°C with constant gentle shaking. Agarose beads were collected by centrifugation, washed with ice-cold RIPA lysis buffer 2 times and PBS 2 times, then boiled in 2X Laemmli sample buffer for denaturation of proteins. The immunoprecipitated protein was used for immunoblotting.

### *In vivo* Insulin and Leptin Signaling

For insulin signaling experiments, mice were intra-peritoneally injected with 10 U/kg body weight of human insulin (Humulin R, Eli Lilly Corp). Ten minutes after injection, gastrocnemius muscles were dissected and frozen in liquid nitrogen for analysis of the insulin signaling proteins as we described [44]. The site-specific phosphorylation (Tyr-612) of Insulin receptor substrate 1 (IRS-1) was measured by immunoblotting with the specific antibodies (Invitrogen). For the measurement of whole phosphorylation of IRS-1, muscle lysates were immunoprecipitated with IRS-1 (Upstate) antibodies, and followed by immunoblotting with phosphotyrosine 4G10 antibody (Upstate). PKB/Akt serine phosphorylation (Ser-473) was measured by immunoblotting with the specific antibody (Upstate). For leptin signaling experiments, mice were intraperiponeally injected with leptin (3 mg/kg body weight) and euthanized 30 minutes later. Gastrocnemius, Soleus and EDL muscles were quickly removed and frozen in liquid nitrogen. Phosphorylation of AMPK (Thr172) and its downstream target, acetyl CoA carboxylase (ACC-Ser79) was measured by immunoblot analysis using phospho-specific antibodies (Cell Signaling Technology, Beverly, MA).

### Hyperinsulinemic-euglycemic Clamps

Hyperinsulinemic-euglycemic clamp experiments were conducted as we previously described [15], [45], [46]. Mice were implanted with indwelling catheters (Micro-Renathane Tubing, MRE-025, Braintree Scientific Inc.) in the right jugular veins. After a 5-day recovery, mice were used for clamp experiments. Mice were fasted overnight (12 hours). After an initial 5 µCi bolus, [3-^3^H]-glucose (Perkin Elmer, Boston, MA) was infused at 0.05 µCi/min for 2 hours to measure basal glucose production during the basal stage. After the basal period, a two-hour hyperinsulinemic-euglycemic clamp was conducted with a primed and continuous infusion of human insulin (Humulin, Eli Lilly, Indianapolis, IN) at a rate of 2.5 mU/kg/min, coupled with a variable infusion of 40% glucose to maintain blood glucose concentrations at 6 mM. [3-^3^H]-glucose (10 µCi bolus, followed by 0.1 µCi/min) was infused throughout the 2-hour clamp period. Blood glucose levels were monitored via tail bleed every 5 minutes in the 1^st^ hour of clamp stage to achieve stable blood glucose levels and every 10 minutes until the end of the 2-hour clamp to maintain constant blood glucose levels. During the last 45 min before the end of clamp period, a bolus of 10 µCi 2-deoxy-D-[1-^14^C]-glucose (Perkin Elmer) was administrated for measuring tissue specific glucose uptake. At the end of the experiments, mice were euthanized, and tissues were removed and immediately frozen in liquid nitrogen for analysis of 2-deoxy-D-[1-^14^C]-glucose content. The rate of whole-body glucose turnover was estimated using a continuous infusion of [3-^3^H]-glucose at 0.1 µCi/min. Hepatic glucose production was calculated by subtracting glucose infusion rate from glucose turnover rate. Tissue-specific glucose uptake was calculated as previously described [45].

### Enzyme Activities

To measure isoform-specific activity of AMPK, α2AMPK was immunoprecipitated with the antibody (Santa Cruz, CA) specific to its α2 catalytic subunit and protein-G. Kinase activity was determined using synthetic SAMS peptide [47]. Citrate synthase and β-hydroxyacyl-CoA activities were determined spectrophotometrically using skeletal muscle homogenates (20-fold dilution) prepared in 0.1 mol/l KH2PO4/Na2PHO4 and 2 mmol/l EDTA, pH 7.2, as previously described [48].

### Total RNA Extraction and Quantitative RT-PCR

Total RNA was extracted using the Tri Reagent kit (Molecular Research Center, Cincinnati, OH), according to the manufacturer’s protocol. The expression of genes of interest was assessed by quantitative RT-PCR (ABI Universal PCR Master Mix, Applied Biosystems, Foster City, CA) using a Stratagene Mx3000p thermocycler (Stratagene, La Jolla, CA), as we previously described [15]. The primer and probe pairs used in the assays were purchased from Applied Biosystems.

### Muscle Histology

Muscle histology and neutral lipid staining were performed as described [49]. Briefly, muscle was frozen in pre-chilled isopentane, placed in OCT, and cut into 10 µm thick sections. Cryosections were stained with either hematoxalin and eosin for muscle morphology or with Oil Red O (ORO) for intramyocellular neutral lipid conent. Images were visualized using a Leica microscope (DM4000B; Leica Microsystems).

### Statistics

All data are expressed as Mean ± SE. Differences between groups were analyzed for statistical significance by one-way or two-way ANOVA followed by Bonferroni or Fisher’s probable least-squares difference *post hoc* test as appropriate.

## Supporting Information

Figure S1 SOCS3 mRNA is elevated in soleus muscle of DIO and *ob/ob* mice. Total RNA was isolated from soleus and SOCS3 RNA levels were measured by quantitative real-time RT-PCR. Data are expressed as mean ± SE, n = 6–8. *p<0.05. A.U.: arbitrary units.
(TIF)

Figure S2 General morphology (**A**) and intramycellular triacylglyerol (IMTG) content (**B**) of skeletal muscle from MCK/SOCS3 and control mice. H&E staining and oil red O staining of skeletal muscle were used to determine the general morphology and IMTG, respectively, and were conducted as described in Materials and Methods.
(TIF)

Figure S3 Quantitation of the western blots of AMPK and ACC phosphorylation. The blots were quantitated with a Li-COR Odyssey Infrared Imager system. Data are expressed as mean ± SE. *p<0.05. A.U.: arbitrary units.
(TIF)

Figure S4 Body weight development of male MCK/SOCS3 and control mice fed a low fat chow (LF, left panel) or high fat diet (HF, right panel).
(TIF)

Table S1 Plasma glucose and insulin levels during the hyperinsulinemic-euglycemic clamp period.
(DOC)
