# Supplementary figures and images for: Regulation of Insulin and Leptin Signaling by Muscle Suppressor of Cytokine Signaling 3 (SOCS3)
Source: PLoS One. 2012 Oct 24;7(10):e47493. doi: 10.1371/journal.pone.0047493 (PMC3480378; doi:10.1371/journal.pone.0047493)

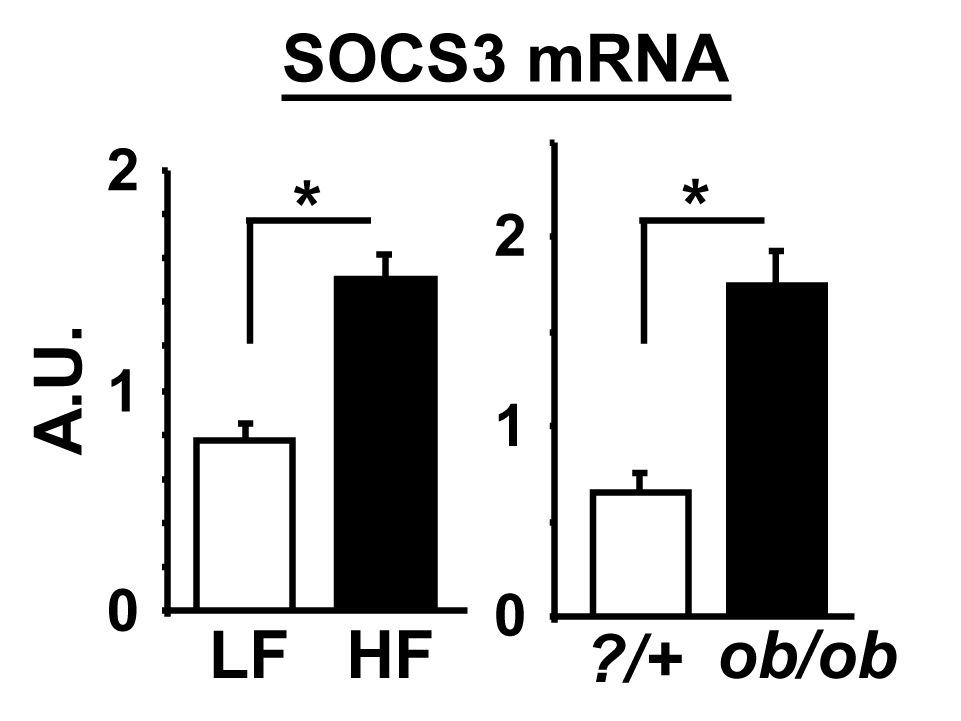

Supplement: Figure S1 — SOCS3 mRNA is elevated in soleus muscle of DIO and ob/ob mice. Total RNA was isolated from soleus and SOCS3 RNA levels were measured by quantitative real-time RT-PCR. Data are expressed as mean ± SE, n = 6–8. *p<0.05. A.U.: arbitrary units. (TIF) [file pone.0047493.s001.tif]

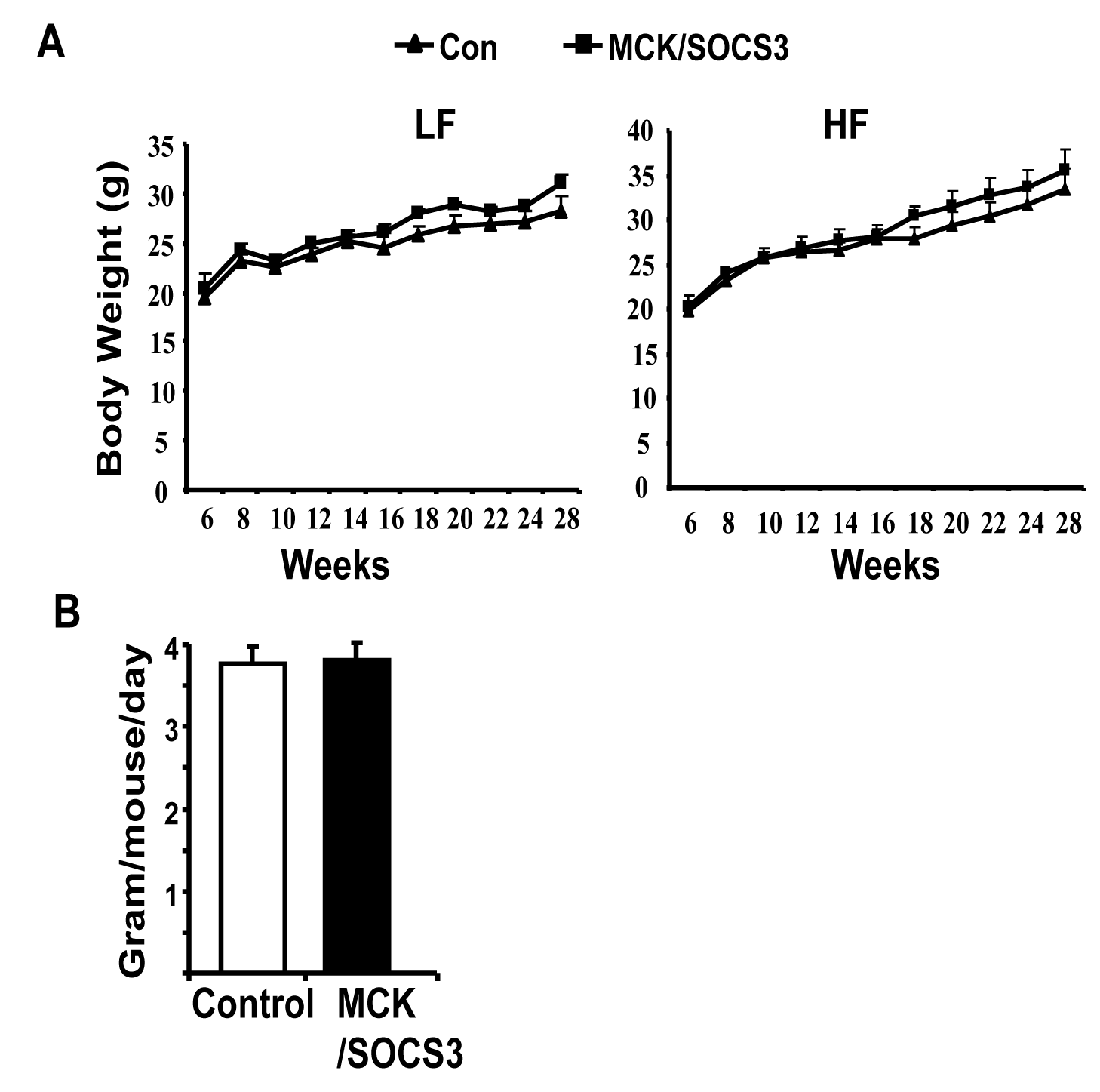

Supplement: Figure S2 — General morphology (A) and intramycellular triacylglyerol (IMTG) content (B) of skeletal muscle from MCK/SOCS3 and control mice. H&E staining and oil red O staining of skeletal muscle were used to determine the general morphology and IMTG, respectively, and were conducted as described in Materials and Methods. (TIF) [file pone.0047493.s002.tif]

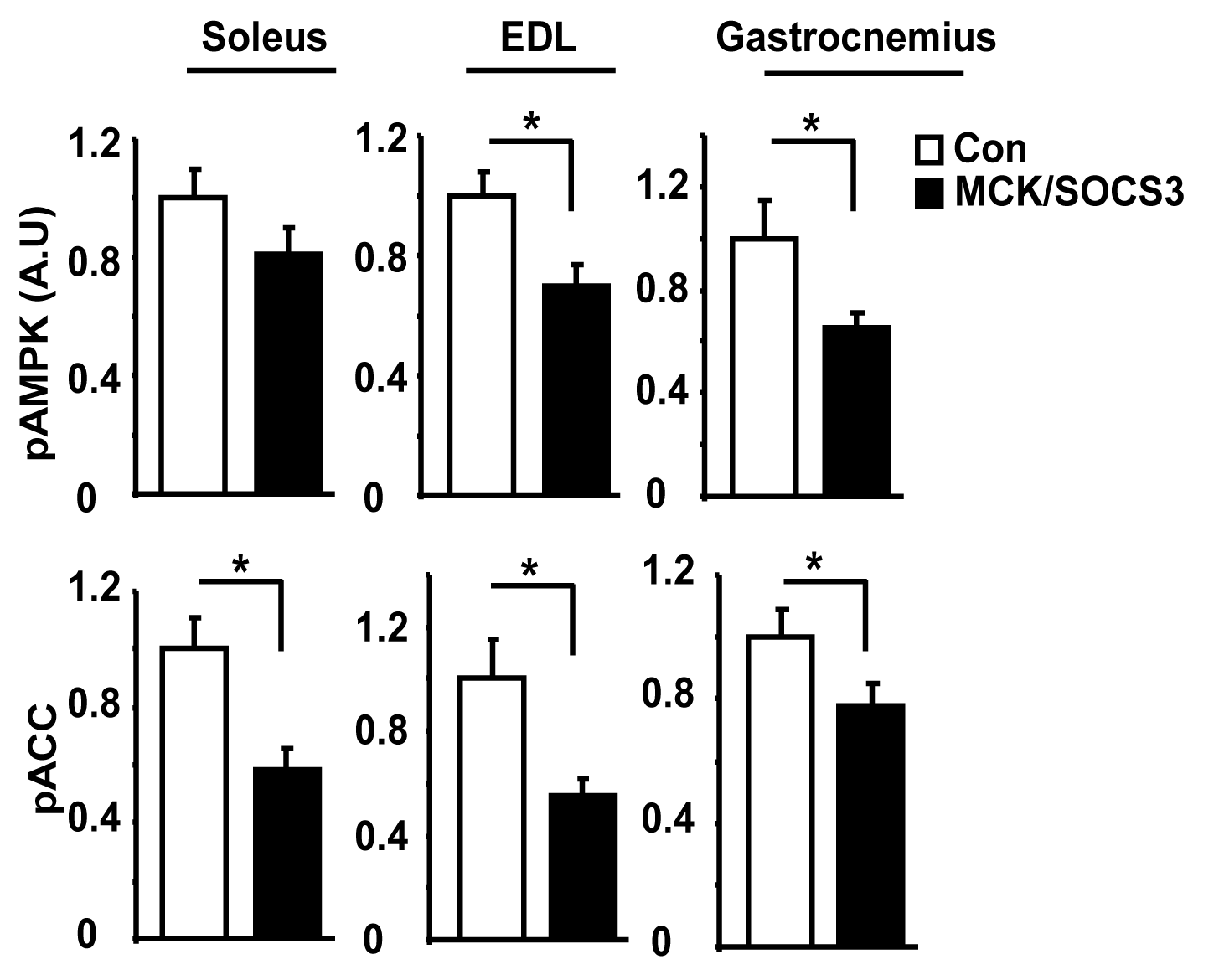

Supplement: Figure S3 — Quantitation of the western blots of AMPK and ACC phosphorylation. The blots were quantitated with a Li-COR Odyssey Infrared Imager system. Data are expressed as mean ± SE. *p<0.05. A.U.: arbitrary units. (TIF) [file pone.0047493.s003.tif]

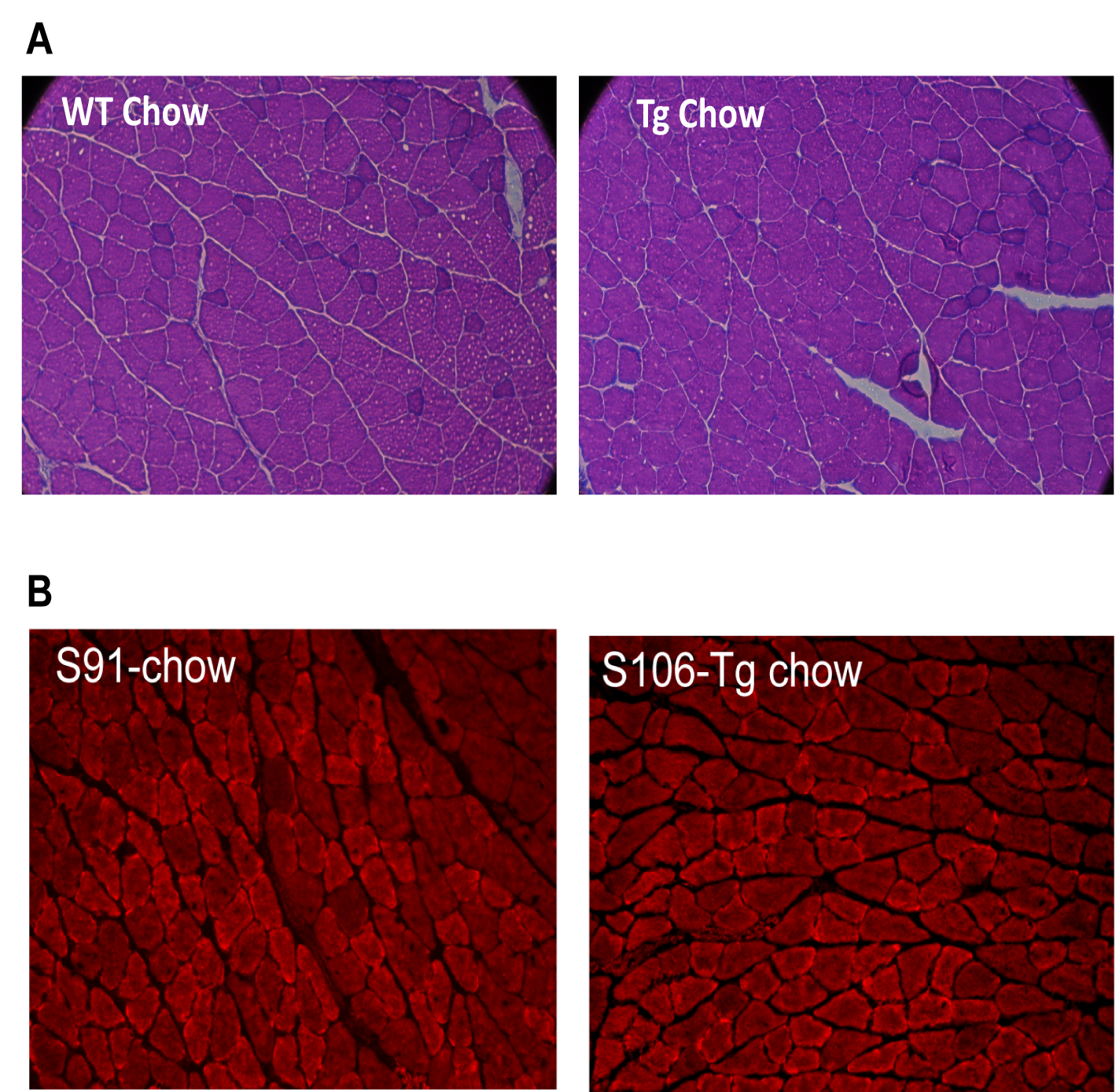

Supplement: Figure S4 — Body weight development of male MCK/SOCS3 and control mice fed a low fat chow (LF, left panel) or high fat diet (HF, right panel). (TIF) [file pone.0047493.s004.tif]
