# Supplementary material for: Regulation of Insulin and Leptin Signaling by Muscle Suppressor of Cytokine Signaling 3 (SOCS3)
Source: PLoS One. 2012 Oct 24;7(10):e47493. doi: 10.1371/journal.pone.0047493 (PMC3480378; doi:10.1371/journal.pone.0047493)
Supplement: Table S1 — Plasma glucose and insulin levels during the hyperinsulinemic-euglycemic clamp period. (DOC) [file pone.0047493.s005.doc]

Supplement Table 1. Plasma glucose and insulin levels during the hyperinsulinemic-euglycemic clamp period

|  | Plasma glucose (mM) | Plasma insulin (pM) |
| --- | --- | --- |
| Control | 6.7 ± 0.5 | 528 ± 59 |
| MCK/SOCS3 | 6.9 ± 0.7 | 535 ± 72 |
